# Microbiological screening tests for SARS-CoV-2 in the first hour since the hospital admission: A reliable tool for enhancing the safety of pediatric care

**DOI:** 10.3389/fped.2022.966901

**Published:** 2022-09-06

**Authors:** Giuseppe Vetrugno, Simone Grassi, Francesco Clemente, Francesca Cazzato, Vittoria Rossi, Vincenzo M. Grassi, Danilo Buonsenso, Laura Filograna, Maurizio Sanguinetti, Martina Focardi, Piero Valentini, Al Ozonoff, Vilma Pinchi, Antonio Oliva

**Affiliations:** ^1^Section of Legal Medicine, Department of Health Surveillance and Bioethics, Fondazione Policlinico Universitario A. Gemelli IRCCS, Università Cattolica Del Sacro Cuore, Rome, Italy; ^2^Section of Forensic Medical Sciences, Department of Health Sciences, University of Florence, Florence, Italy; ^3^Section of Legal Medicine, Department of Interdisciplinary Medicine, Bari Policlinico Hospital, University of Bari, Bari, Italy; ^4^Department of Woman and Child Health and Public Health, Fondazione Policlinico Universitario A. Gemelli IRCCS, Rome, Italy; ^5^Department of Diagnostic and Interventional Radiology, Molecular Imaging and Radiotherapy, PTV Foundation, “Tor Vergata” University of Rome, Rome, Italy; ^6^Laboratory of Microbiology, “A. Gemelli” Hospital, Catholic University of the Sacred Heart, Rome, Italy; ^7^Precision Vaccines Program, Division of Infectious Diseases, Boston Children's Hospital, Boston, MA, United States; ^8^Department of Pediatrics, Harvard Medical School, Boston, MA, United States

**Keywords:** risk management, SARS-CoV-2, COVID-19, hospital-acquired infection, RT-PCR

## Abstract

**Introduction/purpose:**

Since a significant proportion of SARS-CoV-2 infections occur within healthcare facilities, a multidisciplinary approach is required for careful and timely assessment of the risk of infection in asymptomatic patients or those whose COVID-19 diagnosis has not yet been made. The aim of this study was to investigate whether an adaptative model based on microbiological testing can represent a valid risk management strategy.

**Material and methods:**

We collected data from the risk management unit database of a 1,550-bed tertiary hospital (Fondazione Policlinico Gemelli IRCCS, Rome, Italy) concerning pediatric admissions to the Emergency Department (ED) from 1 March 2020 to 31 December 2021. The study period was subdivided in period A and period B according to the technique used for the microbiological screening, respectively reverse-transcription polymerase chain reaction (RT-PCR) and antigen-detection test.

**Results:**

In Period A, 426 children (mean age: 6 years) underwent microbiological screening at the ED. The total number of molecular tests performed was 463. 459/463 tested negative at the molecular test. In Period B, 887 children (mean age: 6 years) underwent microbiological screening in the ED. The total number of molecular tests performed was 1,154. 1,117/1,154 tested negative at the molecular test. Neither in Period A nor in Period B hospital-acquired SARS-CoV-2 infections were reported.

**Discussion and conclusion:**

Despite high volumes, no cases of hospital-acquired SARS-CoV-2 infection have been reported. SARS-CoV-2 antigen-based tests can be used as a first-line option as they provide rapid results compared to RT-PCR, reducing the risk of infection in ED waiting rooms.

## Introduction

A significant share of SARS-CoV-2 infections are known to occur within healthcare facilities, thus representing simultaneous public health and medico-legal challenges (1–4). Indeed, enhancing safety policies during the pandemic can allow for regular delivery of healthcare services and protect particularly vulnerable populations like immunocompromised and oncological patients (2, 3). In order to avoid nosocomial infections, it is needed a careful and timely assessment of the risk of having been infected in the asymptomatic patients or in those whose COVID-19 diagnosis has not been already made (5–7). This demands a multidisciplinary approach using a combination of anamnestic, clinical, microbiologic, and radiologic data to establish the earliest possible diagnosis (8–11).

In this paper, we describe and evaluate the experience of a 1,550-bed tertiary hospital in Italy, where two different risk assessment policies were adopted during the pandemic. Our aim is to investigate and discuss whether an adaptive model chiefly based on microbiological testing can represent a valid risk management strategy from both a public health and medico-legal perspective.

## Materials and methods

We collected data from the Risk Management Unit of Fondazione Policlinico Gemelli IRCCS, Rome, Italy. This is one of the two central hubs in Rome for pediatric COVID-19 cases since the beginning of the pandemic. Data of interest included: number of children admitted at the Emergency Department (ED) from 1 March 2020 to 31 December 2021; age at admission; results of the molecular test; number of hospital-acquired SARS-CoV-2 infections reported.

The definition of “hospital-acquired SARS-CoV-2 infection” is based on the positive result of the molecular test in patients hospitalized for at least 10 days in the ward, who had previously tested negative on the molecular admission test in ED (12–16).

The study period was subdivided in two sub-periods on the basis of what technique was used for microbiological screening: period A (1 March 2020 – 31 October 2020) and period B (1 November 2020 – 31 December 2021). In period A, amplification of SARS-CoV-2 RNA using reverse-transcription polymerase chain reaction (RT-PCR) was used, while in period B antigen-detection test – SD Biosensor antigen-detection test (South Korea), namely the STANDARD F COVID-19 Ag fluorescent immunoassay (FIA) – was adopted (the results of antigen-tests – positive or negative – were then confirmed through RT-PCR).

In both periods, the PCR test administered to the patients upon admission to the emergency room provided a result within 5 h.

**Figure 1:**
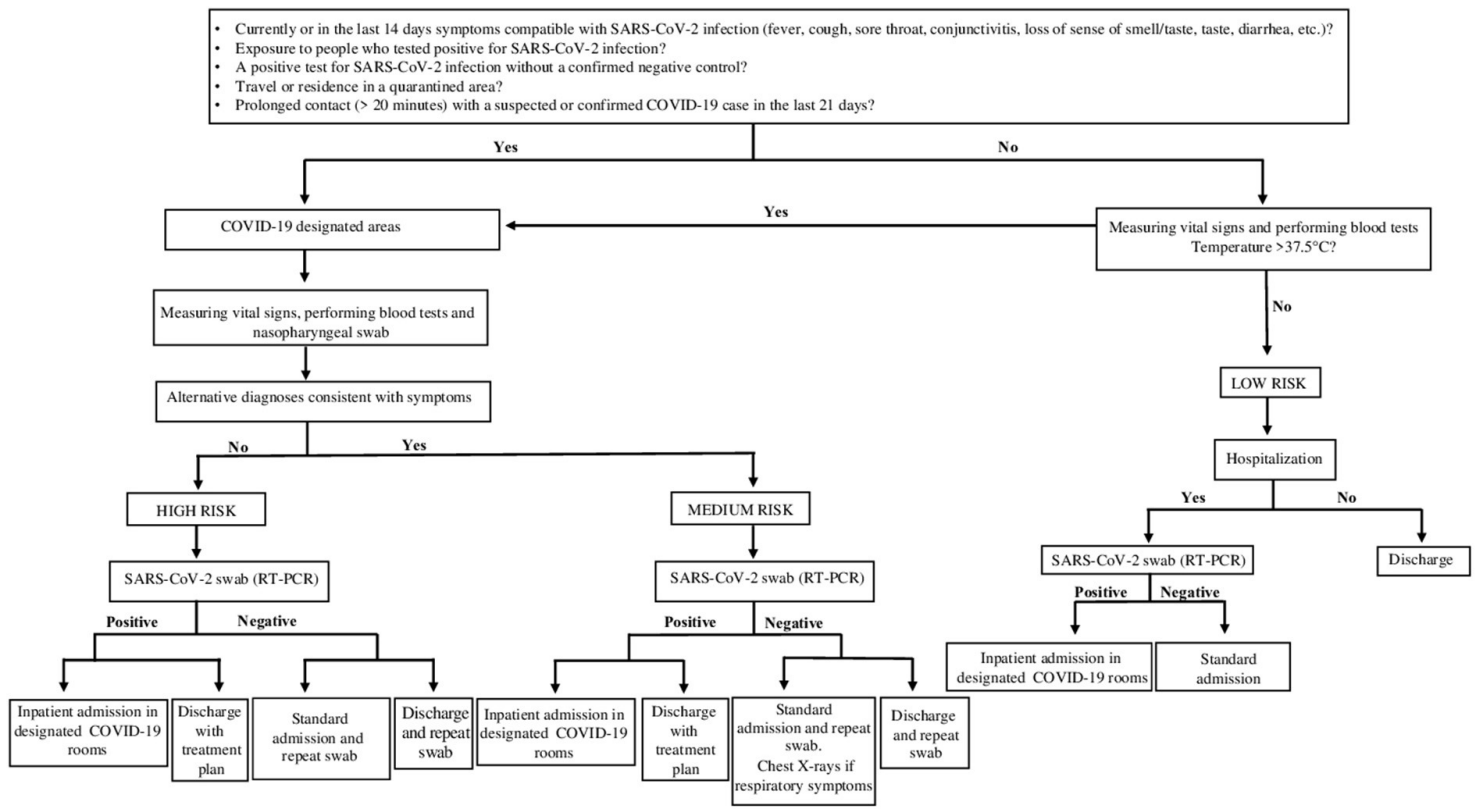
Testing algorithm for diagnosis SARS-CoV-2 infection in patients who presented to the ED from 1 March 2020 – 31 October 2020 (Period A). The standardized screening strategy was molecular swab only (RT-PCR). Waiting for the results of the molecular swab (RT-PCR), patients according to their clinical and epidemiological characteristics were divided into corresponding risk classes (High Risk; Medium Risk; Low Risk).

**Figure 2:**
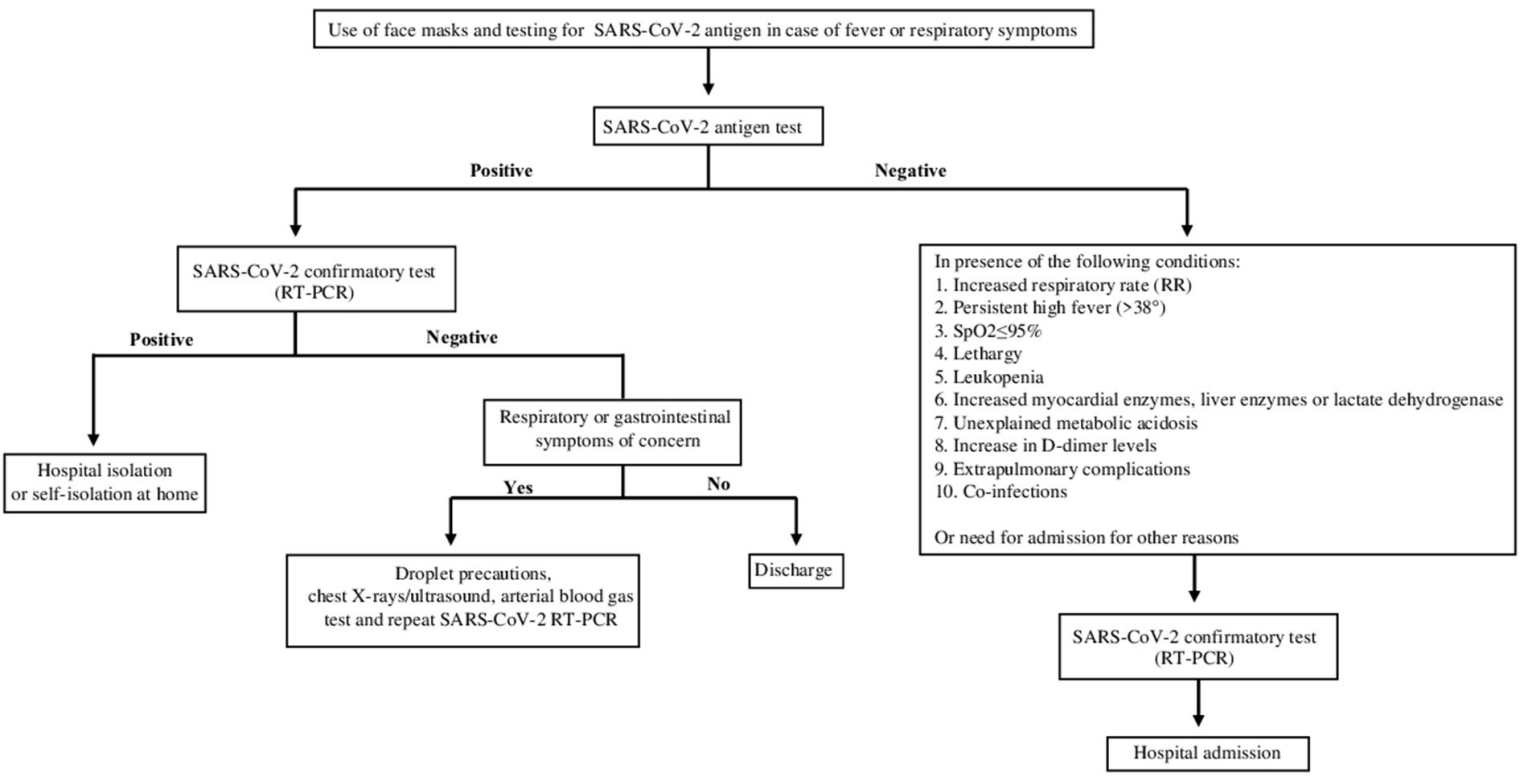
Algorithm for diagnosis SARS-CoV-2 infection in patients who presented to the ED from 1 November 2020 – 31 December 2021 (Period B). The patients with symptoms suggestive of SARS-CoV-2 infection were tested initially by antigenic-test with the SD Biosensor STANDARD F COVID-19 Ag. Then, patients with a positive antigen result and those with a negative antigen result but with one of the following clinical or laboratory criteria shown in the figure were subsequently evaluated by molecular swab (RT-PCR).

During Period B, antigen testing was performed within the first hour after Emergency Department admission, with a corresponding hospital management of pediatric population flow to limit intra-hospital contagion from the Emergency Department (ED) to pediatric ward. The decision algorithms adopted in Period A and Period B are reported, respectively in Figures 1, 2.

Elective hospitalizations were allowed in no-COVID pediatric wards following a mandatory negative result of the PCR test performed in the previous 48 h. Since 7 July 2021, the risk management procedure requires hospitalized patients to repeat the PCR test every 5 days until discharge. This protocol is also the same for the parents of children admitted to the ward.

Hospitalized patients who tested positive were placed in specific respiratory isolation wards at negative pressure, separated from the remaining hospitalization areas for non-COVID children. Furthermore, the medical and nursing teams were also separated, with a staff dedicated exclusively to confirmed positive COVID-cases. The First Aid team integrated the staff of the pediatric COVID-wards.

In period A, waiting for the results of the molecular swab, the patients' assignment of risk class was also supported by clinical and epidemiological characteristics. Indeed, patients were dislocated in areas/paths specific for the risk class depending on the positive response of specific parameters including: presence in the last 14 days of symptoms compatible with SARS-CoV-2 infection: fever, cough, sore throat, conjunctivitis, loss of smell, taste, diarrhea, etc.; family members with coronavirus positive swab; area of residence in quarantine; prolonged contact (>20 min) with a probable or confirmed case of COVID 19 infection in the past 21 days.

In period B, patients with symptoms suggestive of SARS-CoV-2 infection (fever or chills, cough, congestion or runny nose, loss of taste or smell, shortness of breath or difficulty in breathing, body aches, tiredness or headache, sore throat, nausea, vomiting or diarrhea) were analyzed with antigen-test. Then, patients with a positive antigen result and those with a negative antigen result but with one of the following clinical or laboratory criteria (increased respiratory rate (RR):> 50 (2–12 months),> 40 (1–5 years),> 30 (> 5 years); persistent high fever (> 38 °) for 3–5 days or more than 1 week of illness with no improvement in symptoms; SpO_2_ ≤ 95% at rest; hyporeactivity, lethargy; leukopenia; myocardial enzymes, liver enzymes, lactate dehydrogenase progressively increased; unexplained metabolic acidosis; a significant increase in D-dimer levels; extra-pulmonary complications; co-infection with other viruses and/or bacteria) were subsequently evaluated by molecular swab.

In either case, a pediatric patient was admitted to a non-COVID ward in the absence of a negative response to the molecular test.

## Results

**Figure 3:**
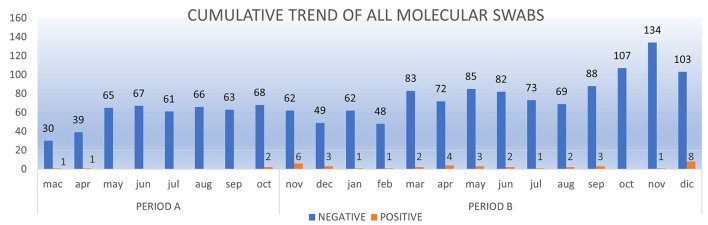
The cumulative trend of all molecular swabs (RT-PCR) from Mach 1, 2020 to December 31, 2020.

**Figure 4:**
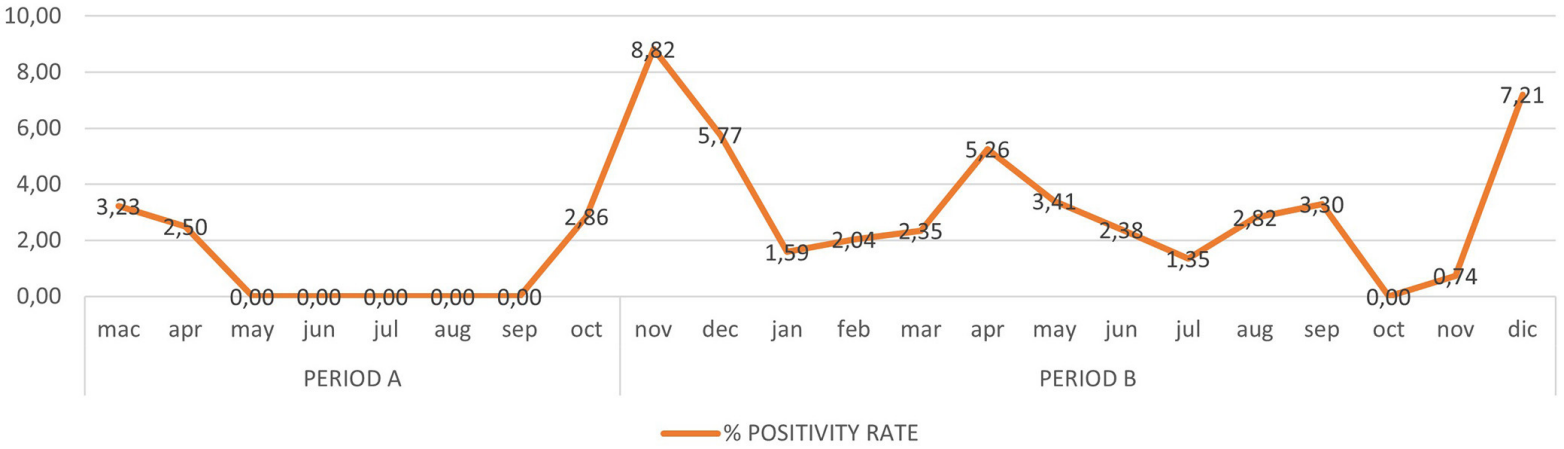
The positivity rate of all molecular swabs (RT-PCR) from Mach 1, 2020 to December 31, 2021.

In Period A, 426 children (mean age: 6 years) underwent microbiological screening at the ED. There were 463 molecular tests performed (according to the risk management protocol for which some patients underwent multiple molecular swabs based on the number of days in hospital). 459/463 molecular tests (99.1%) were negative. In Period B, 887 children (mean age: 6 years) underwent microbiological screening in the ED. These patients received a total of 1,154 molecular tests according to the same risk management protocol, and 1,117/1,154 (96.8%) tested negative at the molecular test. Neither in Period A nor in Period B hospital acquired SARS-CoV-2 infections were reported among the tested children. The cumulative trend of all molecular swabs and the positivity rate are shown respectively in Figures 3, 4.

## Discussion

The aim of this study was to describe and discuss the decisional algorithms used for early identification of SARS-CoV-2-infected children who arrived at our ED.

Despite the high volumes, no cases of pediatric hospital-acquired SARS-CoV-2 infection was reported during the study period. This outcome becomes more important if we evaluate period B. Indeed, while in period A the Italian government imposed a national lockdown (starting 9 March 2020) (17) with a total closure of schools and universities (since 5 March 2020), during period B the second phase of national lockdown (from 2 November 2020 to 27 March 2021) did not require the closure of nursery schools and primary schools (up to 12 years) (18). Furthermore, vaccination prophylaxis, as an additional element of protection against restrictive lockdown measures, had not yet begun to protect both pediatric and adult patients.

In our opinion, the two most important criteria to be used to evaluate risk assessment algorithms are accuracy/reliability and the time required for test results.

From a methodological point of view, RT-PCR is very accurate (Allplex TM SARS-CoV-2 Assay Seegene: Sensitivity 95.2%–Specificity 98.9%) (19) but the results are not available for several hours with the consequent risk of increased transmission (20–22). As an alternative screening method, SARS-CoV-2 antigen-based tests can significantly reduce this time, especially in crowded settings such as a full emergency room where SARS-CoV-2 infection must be confirmed as soon as possible (23–25). Although the antigen test is less sensitive than RT-PCR, it is highly specific and, more importantly, can return a result within 15–30 min (25–27). This is possible thanks to lateral flow technology, which allows identification and visualization of the SARS-CoV-2 antigen as a reactive band for immunoassay on a compact handheld device (19, 28). However, negative results from this method due to the low sensitivity cannot confidently exclude SARS-CoV-2 virus infection and thus results must be verified by further RT-PCR test (29–34). As reported by Menchinelli et al. (35) in an ED, antigen-positive or antigen-negative results must be confirmed subsequently by RT-PCR testing both in patients with a low (<10%; including patients asymptomatic or symptomatic for more than 7 days after symptom onset) and in patients with a high (> 10%; including symptomatic patients within 7 days of symptom onset) probability of testing positive.

Hence, from a public health perspective, the best option is that introduced during Period B: using the antigen test as an initial screen in order to obtain the results within an hour and, in case of a positive result, isolate the patient while waiting for result of a confirmatory RT-PCR. Indeed as noted above, another determinant of the quality of a safety protocol in this context is timeliness, since the early isolation of at-risk patients can avoid a significant spread of the infection within hospital departments. Moreover, the fact that antigen test is associated with lower sensitivity than RT-PCR is only a relative limitation, since in a pandemic context the prevalence of the infection is relatively high and thus the positive predictive value of the microbiological testing is increased.

As reported by Mönckel et al. (36) antigenic test (AGTEST) among symptomatic patients in the ED is useful for early identification of COVID-19, but for patients with negative antigen test this result must be confirmed by molecular test (RT-PCR). However, it was observed that when the prevalence of SARS-CoV-2 infection rises, the positive predictive value increases too.

## Conclusion

In conclusion, our experience shows the effectiveness of a screening strategy based on rapid antigen testing in children initially assessed in the pediatric ED in order to optimize patient flow from the ED to the optimal inpatient wards. The strategy was both timely and safe, since no cases of pediatric hospital acquired SARS-CoV-2 infections were reported. Further studies will be needed to understand how this procedure can be applied with future variants of concerns and a higher vaccination coverage in children.

### Limitations

Our study has some limitations to acknowledge. First, it is a retrospective study. Secondly, we could not determine the number of possible infections acquired in children while attending the pediatric ED and then discharged at home, since no follow-up data were collected for this group of children. Another relevant aspect is the increased cost of testing caused by the introduction of antigen-test screening. During our study period, the economic cost was a variable of relatively minor relevance because in that phase of the pandemic the regional health system covered all the costs for microbiological testing, thus this protocol had no direct costs for the hospital. However, if economic aspects must be considered, a modification to the protocol which might minimize cost is to conduct PCR test only on those antigen-negative patients that are at risk for other (clinical, epidemiological) reasons. This would maximize cost-benefit and reduce the overall number of tests required.

## Data availability statement

The raw data supporting the conclusions of this article will be made available by the authors, without undue reservation.

## Author contributions

All authors listed have made a substantial, direct, and intellectual contribution to the work and approved it for publication.

## Funding

This paper was supported by Università Cattolica del Sacro Cuore, Linea D.3.1 n. R4124501052 (Recipient: AO).

## Conflict of interest

The authors declare that the research was conducted in the absence of any commercial or financial relationships that could be construed as a potential conflict of interest.

## Publisher's note

All claims expressed in this article are solely those of the authors and do not necessarily represent those of their affiliated organizations, or those of the publisher, the editors and the reviewers. Any product that may be evaluated in this article, or claim that may be made by its manufacturer, is not guaranteed or endorsed by the publisher.
